# Challenges Faced by Family Caregivers of Individuals Living with Dementia in Japan During the COVID-19 Pandemic

**DOI:** 10.3390/nursrep14040285

**Published:** 2024-12-07

**Authors:** Toshiko Tsuyuki, Takeshi Asai, Erina Kurosaki, Atsushi Nakamura, Kaori Kishi, Fumi Takeda

**Affiliations:** 1 Motion SciTech Research Center, Grand Chariot Takezono 402, Takezono 2-7-8, Tsukuba-City 305-0032, Ibaraki, Japan; qqcv6wfd@oasis.ocn.ne.jp; 2 Institute of Health and Sports Sciences, University of Tsukuba, Tennodai 1-1-1, Tsukuba-City 305-8574, Ibaraki, Japan; takeda.fumi.fe@u.tsukuba.ac.jp; 3 Advanced Research Initiative for Human High Performance (ARIHHP), Faculty of Health and Sport Sciences, University of Tsukuba, Tennodai 1-1-1, Tsukuba-City 305-8574, Ibaraki, Japan; kurosaki.erina.gf@un.tsukuba.ac.jp; 4 Information Media Center, Hiroshima University, Kagamiyama 1-4-2, Higashi Hiroshima-City 739-0046, Hiroshima, Japan; nakamura@an-pan.org; 5 Faculty of Social Welfare, Iwate Prefectural University, Sugo 15252, Takizawa-City 020-0061, Iwate, Japan; kaori_k@iwate-pu.ac.jp

**Keywords:** dementia, individuals living with dementia, family caregiving, nursing, formal support, informal support, COVID-19

## Abstract

Background/Objectives: This study investigates the challenges faced by family caregivers of individuals with dementia in Japan, particularly in the context of the COVID-19 pandemic. Methods: We conducted a cross-sectional survey of 500 family caregivers of patients with dementia. Results: 56.4% of caregivers reported an increased caregiving burden, primarily due to extended caregiving hours, reduced access to public services, and restrictions on social interactions. This study found a strong preference for formal support, with 75.4% of caregivers desiring access to more comprehensive services. However, 19.4% of dementia patients were not utilizing any public services, largely due to a mismatch between available services and caregivers’ actual needs, as well as societal resistance to inviting formal support into the home. Informal support systems, such as dementia family groups and cafes, were well-known, but participation rates remained low (5.4% and 5.8%, respectively), despite the potential benefits for reducing stress and providing emotional support. Key barriers included privacy concerns, reluctance to engage, and logistical challenges such as inconvenient access and time constraints. Conclusions: To mitigate the above challenges, this study recommends expanding telemedicine and remote support services, improving awareness of available resources, and offering flexible, tailored solutions to meet diverse caregiving needs. Additionally, increasing financial support, enhancing public recognition of caregiver roles, and providing psychological counseling and stress management programs are essential to alleviating both the emotional and economic burdens placed on family caregivers during the pandemic.

## 1. Introduction

The global demographic shift toward an aging population has led to a significant rise in the number of individuals diagnosed with dementia, creating a pressing need for effective caregiving solutions [1,2,3,4,5]. Caregiving for individuals living with dementia encompasses physical, emotional, and economic challenges and is further complicated by difficulties in accessing high-quality medical and caregiving services [6,7]. Significant advancements have been made in research and caregiving innovation for dementia, yet social stigma and misconceptions continue to create challenges for both individuals with dementia and their family caregivers [8]. Consequently, continued efforts and support are needed to further address these complex issues effectively [9].

Japan, with its rapidly aging population, mirrors these global challenges and is witnessing a dramatic increase in dementia cases [10,11,12,13,14,15,16,17]. The scarcity of trained caregiving professionals raises concerns about the quality of care provided [12], while the high financial burden of dementia caregiving further strains families. Additionally, inadequate integration of medical and caregiving services complicates access to necessary support for individuals living with dementia and their families [18]. Deep-seated societal misunderstandings and stigma surrounding dementia lead to discrimination and social isolation for those affected [19,20,21].

In response to these issues, Japan’s Ministry of Health, Labour, and Welfare introduced the New Orange Plan in 2015, aimed at educating the public about dementia and supporting those in need of care. This plan emphasizes the perspectives of individuals living with dementia and their caregivers, providing a range of caregiving support services and social assistance [22]. Despite these efforts, research has identified substantial challenges in both formal and informal support systems for dementia care in Japan. For instance, studies on person-centered care in group homes indicate that while staff may recognize the importance of empathy in caregiving, they often struggle to consistently express it in practice [23]. Additionally, surveys of residential care facilities have identified a wide range of challenges experienced by family caregivers, including physical, psychological, and economic strains and social isolation [24,25,26,27,28].

The onset of the COVID-19 pandemic in late 2019 introduced new complexities, affecting various aspects of caregiving, from increased infection risks to heightened caregiving burdens [25,29,30,31]. Despite the disproportionate risks during the pandemic, a few comprehensive surveys have been conducted to assess the situation of individuals living with dementia and their family caregivers [32,33,34,35,36]. Research on the challenges faced by family caregivers during this unprecedented period is particularly scarce. Conducting a survey under these extraordinary circumstances could provide valuable insights into the current challenges and needs of family caregivers, potentially informing improved support and policy measures.

In Japan, formal and informal support systems are crucial for dementia care, yet research on their challenges remains limited. Investigating the state and issues of these support systems during the COVID-19 pandemic can contribute to solving problems and improving the quality of family caregiving. Therefore, we conducted a cross-sectional survey among family caregivers of individuals living with dementia in Japan to examine the current state and challenges of formal and informal support, explore caregiving issues and solutions, and gather foundational data for future improvements and policy measures.

## 2. Materials and Methods

### 2.1. Participants

To comprehensively investigate the circumstances of and support mechanisms for family caregivers of individuals living with dementia amidst the pandemic, we conducted an internet-based questionnaire survey (a *web survey*) during the fiscal year 2021 [37,38]. The survey participants were scattered across all regions of Japan, from Hokkaido to Okinawa prefectures. This survey was conducted following a screening survey of 10,000 individuals, in collaboration with a contracted research company, using a master sample of 500 internet monitors who agreed with the survey’s purpose. Participants included patients diagnosed with dementia at levels above mild cognitive impairment (MCI) by medical institutions. Participants in this study did not receive any financial or other forms of compensation.

### 2.2. Sampling Method

The sex ratio (*male–female*) of the study population was approximately equal, with 50 participants randomly selected from all age groups except those ≥70 years. To ensure homogeneity in respondent demographics, we implemented random adjustments within distinct age groups (20–30s, 40s, 50s, 60s, and 70s and above), resulting in an approximate distribution of 100 respondents per age category [2]. While ensuring equal age distribution and gender ratio among the sample can lead to drawbacks, including reduced external validity and resource inefficiency, our study prioritized benefits such as securing representativeness and reducing bias. Our sample comprises an equal distribution of men and women; however, it is not fully representative of the general Japanese population. Therefore, findings should be interpreted within the context of this specific sample.

### 2.3. Survey Content

To alleviate the respondents’ burden, we streamlined and simplified the survey questionnaire, incorporating 25 items from the “Survey on the Actual Situation of Caregiving by General Citizens for Family Members with Dementia” (Table 1) [17,23,39].

### 2.4. Data Collection

The online survey was conducted in October 2021, considering the persistent impact of the pandemic that commenced in 2019. To facilitate comparison and analysis with traditional situational surveys, we employed, to the extent possible, similar questionnaire items as used in conventional surveys. The execution of the web survey was entrusted to Survey Research Center, Inc., a reputable private research firm. The respondents were explicitly assured that the survey results would be exclusively employed for academic purposes, with individual responses safeguarded from disclosure, ensuring the confidentiality of their data. They were only requested to provide information about their residential location, age, and gender.

### 2.5. Data Analysis

The responses garnered through the questionnaire survey were systematically aggregated by item, facilitating the calculation of response frequencies and percentages. A chi-square test was employed for goodness of fit, with the pre-established significance level (*p*) set at 5% (*p* < 0.05) to assess the statistical significance of response percentages. The *p*-value was expressed using exponential notation, as necessary.

### 2.6. Definitions of Formal and Informal Support

In this study, formal support was defined as assistance provided by public organizations or specialized institutions. Typically, it involves systematically and institutionally structured services, encompassing support from medical facilities, care institutions, and social welfare agencies. Contrastingly, informal support is assistance rendered by individuals within the general populace and members of the local community. Unlike formal support, it does not involve intervention from public institutions or professionals; rather, it is rooted in personal relationships and community ties. This category predominantly includes support from family members, friends, neighbors, and local volunteers.

Within this study’s framework, support was broadly categorized into two distinct groups: “formal support” and “informal support”, encompassing services covered and not covered by long-term care insurance, respectively.

In this study, the “dementia café” referred primarily to a form of informal support operated by family associations, social welfare corporations, and nonprofit organizations. It serves as an information-sharing and exchange salon where individuals living with dementia and their families gather.

### 2.7. Ethics Approval

This research was conducted with the explicit approval of the Sports Science Research Ethics Committee of Tsukuba University (Approval number: Tai 021-11). All participants provided written informed consent to participate in this study and have their data published in the manuscript.

## 3. Results

We obtained responses from 500 individuals (272 males: 54.6%; 228 females: 45.4%), with caregiving experience of individuals living with dementia via a web survey (Table 1) administered in 2021.

Regarding their general familial relationship with the person living with dementia, 48.2% of caregivers identified as mothers, 15.6% as fathers, 12.8% as grandparents, and 10.2% as stepparents. However, among those currently residing with the person living with dementia, caregiving was primarily undertaken by sons or daughters (60.4%), spouses (38.2%), or grandchildren (15.2%), highlighting the closer kinship typically seen in cohabitation settings. Although cohabitation with adult children was prevalent, situations involving cohabitation and interaction with younger generations, such as grandchildren, were also observed.

**Figure 1:**
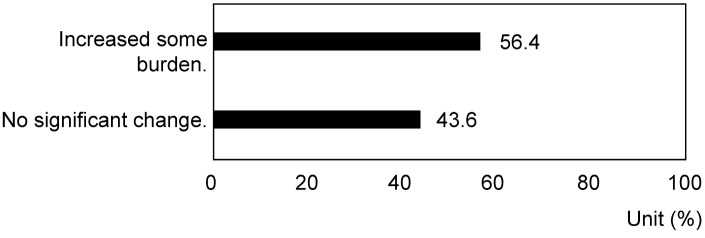
Response rate regarding increased care burden during the COVID-19 pandemic (*p* < 0.01).

Regarding the impact of COVID-19 on caregiving burdens, participants reported an increase in various burdens, with 56.4% indicating some form of increased burden (Figure 1) (*p* = 5.23 × 10^−14^) (exponential notation). While 56.4% of caregivers reported an increased burden during the COVID-19 pandemic, 43.6% did not experience this increase. Although we collected data on various caregiving factors, no significant associations were identified between resilience to increased burden and specific support services or circumstances, such as the use of non-public support. These burdens included increased caregiving time owing to self-quarantine, resulting in a loss of personal free time (27.8%), disruptions in daily routines leading to health issues (24.6%), and for 22.0% of respondents, either the suspension of community activities or the inability to alleviate stress. Free-text responses revealed concerns related to infection control measures, such as “the thoroughness of the person living with dementia’s infection control”, the impact of behavioral restrictions, such as “restrictions on visitors from other prefectures”, and interruptions in service utilization, such as “caregivers from other prefectures being treated as visitors, making it very difficult to receive visits from helpers or care managers”. During the COVID-19 pandemic in Japan, there were government-imposed travel restrictions between prefectures to reduce the spread of infection, which significantly limited essential support services. This led to difficulties in caregivers’ ability to receive visits from helpers or care managers from other prefectures. Additionally, descriptions of changes in the condition of the individual living with dementia, such as “the disruption of the family’s daily routine due to COVID-19, resulting in the rapid progression of dementia”, and the impact on employment, such as “the lack of caregiving support during periods when facility usage was not possible, prevented me from working”, were mentioned.

Regarding caregivers’ preferences for the caregiving environment, 54.2% expressed a preference for “at their familiar home” (*p* = 2.19 × 10^−26^), based on the Ricardian scale of the Four Principles Law (multiple choices allowed). Similarly, 65.8% expressed a preference for “caring for their family at home using welfare services (e.g., visiting services and day care facilities)” (*p* = 1.15 × 10^−14^), 54.4% preferred “entrusting caregiving to professionals in a facility” (*p* = 1.09 × 10^−35^), and 51.6% favored “alternating between a facility and home” (*p* = 1.71 × 10^−44^).

**Figure 2:**
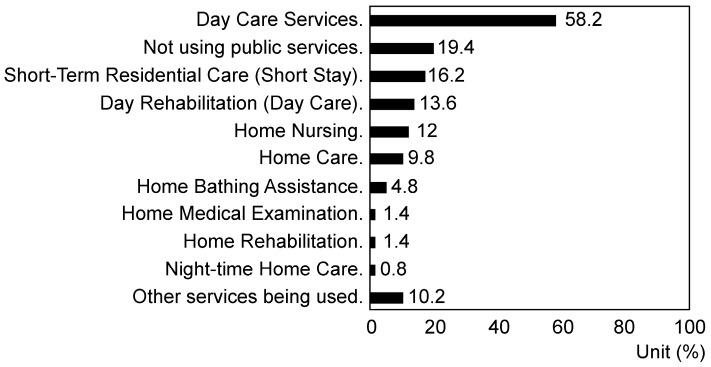
Response rate regarding welfare services currently used by people living with dementia (multiple responses allowed) (*p* < 0.01).

Regarding “Welfare services currently used by the person living with dementia” (multiple choices allowed), 58.2% used day care services most frequently, followed by 19.4% who did not use any public services and 16.2% who used short-stay residential care (short-stay) (*p* = 6.97 × 10^−245^) (Figure 2).

**Figure 3:**
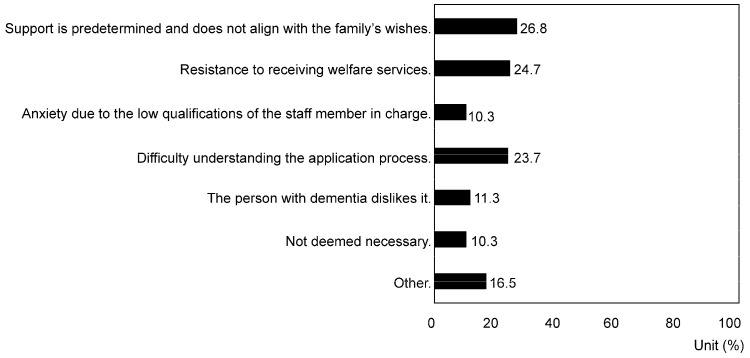
Response rate regarding reasons for not using public services (multiple responses allowed) (*p* < 0.01).

Although a minority (19.4%), some caregivers did not use public services. Their reasons for not utilizing public services included its offerings not aligning with family preferences (26.8%), resistance to using welfare services (24.7%), difficulty in understanding the application process (23.7%), and the reluctance of individuals living with dementia (11.3%) (*p* = 0.0075) (Figure 3).

**Figure 4:**
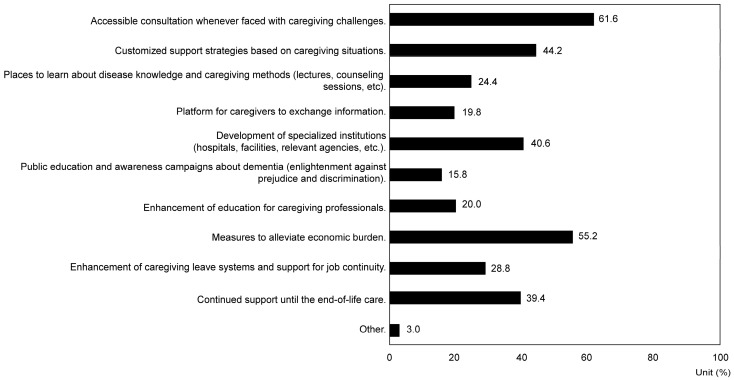
Response rate regarding desired types of public support to continue caregiving (multiple responses allowed) (*p* < 0.01).

Concerning the “Initial consultation source when noticing the person living with dementia’s symptoms”, 32.2% consulted their family physicians, 26.2% family members living with or separately from the person living with dementia, 21.8% dementia specialists, 14.4% welfare-related agencies, 1.6% did not consult anyone, and 0.4% consulted local family support groups (*p* = 6.61 × 10^−72^). Regarding the question, “What kind of public support would be beneficial to continue caregiving?” (multiple choices allowed), 61.6% preferred having access to a consultation service whenever they needed it, 55.2% favored measures to alleviate financial burdens, and 44.2% preferred customized support tailored to their specific caregiving circumstances (*p* = 2.22 × 10^−99^) (Figure 4).

**Figure 5:**
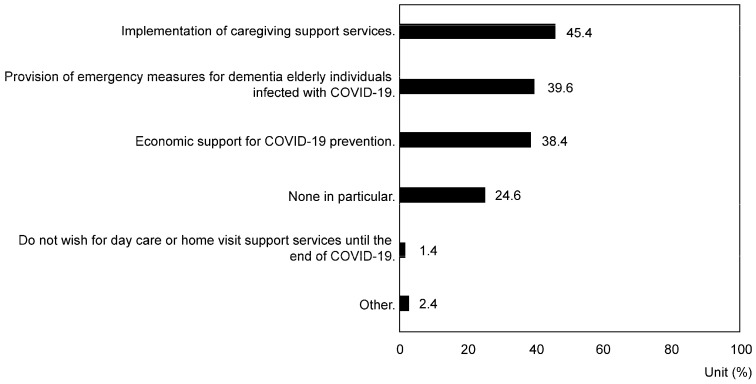
Response rate regarding desired support for the spread of COVID-19 (official and unofficial) (multiple responses allowed) (*p* < 0.01).

In the section titled “Support desired for coping with the spread of COVID-19” (both official and unofficial support, multiple choices allowed), 75.4% of respondents expressed a desire for official support, with 45.4% favoring the implementation of caregiving support services. Emergency measures for COVID-19-infected older individuals living with dementia were favored by 39.6%, and financial support for COVID-19 prevention was desired by 38.4% (*p* = 5.82 × 10^−78^) (Figure 5).

Conversely, regarding “awareness of meeting places for dementia families, such as dementia family groups and dementia cafés”, 60.8% were aware of dementia family groups (*p* = 1.37 × 10^−6^), and 56.0% were aware of dementia cafés (*p* = 0.0073). However, only 5.4% and 5.8% of caregivers participated in dementia family groups and cafés, respectively.

**Figure 6:**
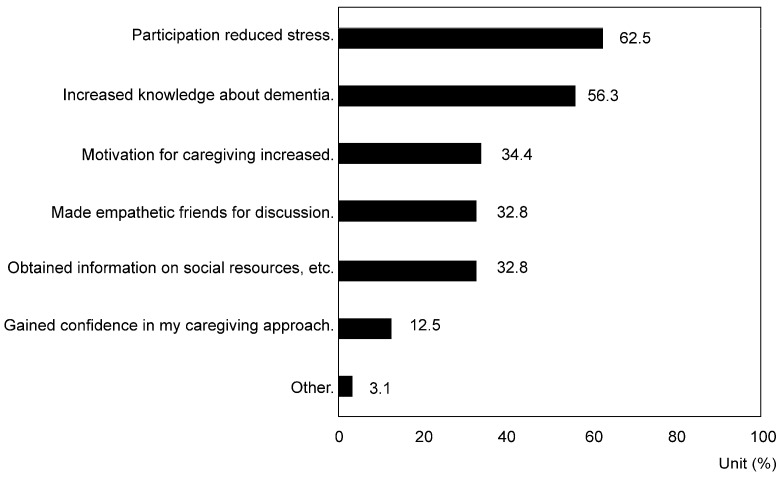
Response rate regarding the effects of participation in dementia family associations and dementia cafés (multiple responses allowed) (*p* < 0.01).

When asked about the “Source of information that led to awareness of meeting places for dementia families” (multiple choices allowed), 40.2% cited comprehensive community support centers, 38.0% mentioned municipal or village public newsletters, and 23.0% referred to daycare services (*p* = 3.93 × 10^−94^). Additionally, when asked about the “Effects of participating in dementia family groups or dementia cafés” (multiple choices allowed), 62.5% reported reduced stress due to participation. In comparison, 56.3% claimed to have gained knowledge about dementia (*p* = 1.81 × 10^−9^) (Figure 6). In contrast, when asked about the “Reasons for being aware of meeting places for dementia families but not intending to participate” (multiple choices allowed), the most common reason was resistance to caregiver gatherings (31.9%), followed by a lack of understanding about the content (28.2%) and not experiencing significant caregiving difficulties (27.3%) (*p* = 1.86 × 10^−19^).

## 4. Discussion

The purpose of this study was to conduct a cross-sectional survey of family caregivers of individuals living with dementia in Japan, investigating the current state and challenges of both public and non-public support and exploring issues and solutions related to family caregiving. The results revealed an increase in caregiver burden during the COVID-19 pandemic, a high diversity of needs for public support, and a low utilization rate of non-public support despite high awareness. The questionnaire survey conducted in this study targeted family caregivers of individuals living with dementia, offering the advantage of rapid data collection [37]. However, caution is necessary owing to potential biases in sampling, the validity of questionnaire items, and respondents’ anonymity [38]. In this survey, respondents’ ages ranged from those in their 20s to those aged ≥70, with nearly equal representation, suggesting minimal sampling bias. In 2016, Japan’s sex distribution of family caregivers aged ≥15 years was as follows: 2.78 million (39.7%) were male, and 4.21 million (60.3%) were female, with females outnumbering males. However, in this survey, the participants’ sex ratio was kept nearly equal to solicit opinions from both men and women equally [39]. Moreover, many questionnaire items were selected based on previous research and were believed to cover various aspects of the targeted phenomenon comprehensively [40,41]. Additionally, computer programs automatically processed respondent data without identifying individuals, ensuring anonymity and objectivity. Consequently, survey results can reasonably be assumed to validly analyze and examine the circumstances and challenges faced by family caregivers depending upon the support environment.

In this survey, 56.4% of caregivers reported an increase in burden related to COVID-19. The main factors included reduced free time due to increased caregiving hours at home (27.8%), interruptions in daily life and resulting health issues (24.6%), and restrictions on community interactions and reduced opportunities for stress relief (22.0%). In open-ended responses, caregivers expressed concerns about infection prevention measures, mobility restrictions, disruption of caregiving services, changes in the condition of dementia patients, and work-related impacts. Studies on the psychological impact of COVID-19 on caregivers have reported increased psychological burden, including heightened anxiety and depression levels due to pandemic-related stress [42]. These findings suggest that the increased caregiving burden due to COVID-19 is primarily caused by increased caregiving hours, the emergence or worsening of health issues, and restrictions on community activities.

Caregivers’ preferred caregiving environments varied widely, including home-based care (54.2%), home-based care with welfare services (65.8%), professional care in facilities (54.4%), and a combination of facility and home care (51.6%). These preferences indicate that caregivers seek flexible support tailored to their individual circumstances rather than one-size-fits-all solutions. Furthermore, professional support is essential for severely affected dementia patients. These preferences significantly impact caregivers’ burden, highlighting the need for specialized support for those with severe dementia.

Regarding the welfare services currently utilized by dementia patients, day care services were the most frequently used (58.2%), followed by short-stay services (16.2%). However, a certain percentage of patients (19.4%) did not utilize any public services. In many developed countries, family caregivers of dementia patients have utilized various services, such as home nursing services, dementia support, day care, and respite care centers, even before the COVID-19 pandemic [1,2,4]. Japanese family caregivers also utilize these services and comprehensive community support centers [13,14,15]. This survey indicates that over 80% of individuals utilize public support, suggesting that a comprehensive care system providing various services is crucial for family caregivers. However, barriers to utilizing public support include a mismatch between the actual support and the needs of dementia patients and caregivers (26.8%) and vague resistance stemming from societal expectations and perceptions about inviting support staff into homes (24.7%) (*p* = 0.007). Thus, support providers must carefully investigate and consider the preferences and needs of dementia patients and caregivers when initiating formal support. It is also important to reduce resistance and anxiety related to receiving support by enhancing societal recognition of dementia patients and caregivers while reducing prejudice. Furthermore, providing detailed information about support facilities and services in an easily understandable and relatable manner through various media, such as newspapers, municipal newsletters, the internet, and social networking services, is also crucial.

As a response to the expansion of COVID-19, 75.4% of caregivers expressed a desire for formal support, specifically for the implementation of support services (45.4%), emergency measures for COVID-19 infection among patients with dementia (39.6%), and economic support for infection prevention (38.4%) (*p* = 5.82 × 10^−78^). American family caregivers desire online support and remote counseling [43], while Canadian caregivers seek remote care, information provision, and respite care services. These data suggest that the primary challenges for caregivers during the pandemic in Japan include reducing infection risk, managing burdens related to infection prevention, addressing service limitations, easing restrictions on facility admission, reducing social isolation and loneliness, and alleviating economic burdens. To address these challenges, Japan’s public support strategy should focus on developing a comprehensive community care system that effectively integrates public and community-based resources to support caregivers. It is necessary to enable family caregivers to receive remote medical services and counseling. Additionally, when access to care facilities and day care centers is restricted, providing temporary home healthcare services and remote rehabilitation programs is crucial. Allowing family caregivers to participate in remote caregiver support groups is also beneficial, especially when face-to-face interactions among caregivers are limited. Moreover, providing psychological counseling services and stress management programs is important to alleviate caregivers’ stress and isolation. At the same time, establishing a proxy caregiver support system to prevent caregiver burnout is necessary [44,45]. To alleviate caregivers’ economic burden, direct subsidies, expanded tax deductions, and support for taking caregiving leave are also important economic support measures.

Although the recognition rate for informal support systems such as dementia family groups and dementia cafes is high in Japan (60.8% and 56.0%, respectively), actual participation rates are extremely low (5.4% and 5.8%, respectively). Participation has demonstrated certain benefits, such as information gathering, stress reduction, and emotional support. For example, one respondent mentioned, “Talking to other families at the dementia family group made me realize that my feelings and actions are normal, which gave me a sense of relief.” On the other hand, some families do not utilize support, citing resistance to discussing personal problems with others, inconvenient transportation, and time constraints. To overcome these challenges, it is important to strictly protect privacy, including personal information and consultation content, and emphasize this protection to provide a safe environment for utilizing support. Additionally, gradually introducing support and reducing resistance to information provision when using support for the first time, while building trust relationships, is also essential. Furthermore, offering support services through online platforms, such as video conferencing and chats, to reduce caregivers’ geographical and time constraints is effective.

## 5. Conclusions

This study aimed to conduct a cross-sectional survey on the current status and challenges of public and non-public support for family caregivers of individuals with dementia residing in Japan and to explore issues related to family caregiving and possible solutions.

According to the analysis of responses from 500 caregivers, the COVID-19 pandemic significantly increased caregiver burdens, specifically through increased caregiving hours, disruption of daily life, and restrictions on community activities. Additionally, barriers to using public support, such as discrepancies between family needs and the support provided, as well as procedural difficulties, were identified. The need for economic support and personalized assistance was emphasized. Furthermore, although the awareness of non-public support was high, its utilization rate was low, highlighting the need for specific measures to enhance engagement and promote the use of these resources.

To improve the utilization rate of public support, it is necessary to simplify application procedures and provide flexible support menus tailored to caregivers’ needs, addressing the procedural difficulties and mismatches in support content faced by family caregivers. Additionally, educational campaigns and enhanced support systems are essential to reduce the psychological resistance caregivers may feel when utilizing support services. Enhancing information dissemination is essential to promote the use of non-public support, especially considering that informal resources, such as dementia cafes and family groups, help reduce stress and facilitate information exchange for family caregivers. Supporting the expansion of these activities is also crucial. In emergencies, such as the COVID-19 pandemic, expanding emergency caregiving support services and introducing economic support measures are important. This includes continuing home-visit care services with thorough infection prevention measures and providing temporary financial assistance to caregivers.

This study has several limitations. First, the survey was limited to caregivers who could use the internet, potentially introducing bias due to the digital divide. Additionally, as a cross-sectional survey, it is difficult to clarify causal relationships. This study also relies on self-reported data from questionnaires, which may be influenced by respondents’ subjectivity and memory bias. Finally, this study is limited to Japan, and caution is needed when generalizing the results to other countries.

Future research should focus on long-term and multinational comparative studies to deepen the understanding of the diverse aspects of family caregiving. Longitudinal studies are needed to clarify causal relationships, and research evaluating the effectiveness of caregiving support measures tailored to specific needs of caregivers should be conducted.

## Figures and Tables

**Table 1:** Questionnaire items concerning “Welfare services currently used by people living with dementia”.

Question Numbers	Question Contents
1	How many years of caregiving experience did the caregiver have?
2	What was the relationship between the caregiver and the person living with dementia?
3	What was the caregiver’s occupation?
4	What was the age of the person living with dementia being cared for?
5	How many family members were currently co-residing with the person living with dementia?
6	What was the degree of independence in the current daily life of the person living with dementia being cared for?
7	What welfare services were currently being used?
8	What were the reasons for not utilizing public services or support?
9	Whom did you consult when you first noticed signs of dementia in the family member?
10	How many hours per week are you involved in caregiving?
11	Do you have any collaborators/partners in caregiving?
12	What kind of public support do you think would be helpful to continue caregiving?
13	Are you aware of dementia family support groups or dementia cafés?
14	Where did you first learn about gatherings for families dealing with dementia?
15	What were your reasons for not considering participation in dementia family gatherings, despite being aware of them?
16	What benefits do you think you have gained from participating in family support groups or dementia cafés?
17	Did your caregiving environment change owing to the spread of the novel coronavirus (COVID-19)?
18	Did your caregiving burden change owing to the spread of the novel coronavirus (COVID-19)?
19	What kind of public support do you hope for in response to the spread of the novel coronavirus (COVID-19)?
20	Do you desire activities by community support organizations in response to the spread of the novel coronavirus (COVID-19)?
21	If caregiving is under the New Orange Plan, is providing care at their familiar home the best option for the family?
22	If caregiving is under the New Orange Plan, is it best to receive public welfare services and provide care at home?
23	If caregiving is under the New Orange Plan, is it best to place the person in a facility by entrusting professional caregivers?
24	If caregiving is under the New Orange Plan, is it best to provide care while moving between a facility and home?
25	If caregiving is under the New Orange Plan, what are the other considerations? (provide specific details)

## Data Availability

The original contributions presented in this study are included in the article, and further inquiries can be directed to the corresponding author/s.

## Public Involvement Statement

We conducted the survey only with participants who had been fully informed about the research content and methodology in advance and had provided their consent to both.

## Guidelines and Standards Statement

This manuscript was drafted against the CHERRIES (Checklist for Reporting Results of Internet E-Surveys, Eysenbach, 2004) for online survey research.
